# Leveraging 3D Printing Capacity in Times of Crisis: Recommendations for COVID-19 Distributed Manufacturing for Medical Equipment Rapid Response

**DOI:** 10.3390/ijerph17134634

**Published:** 2020-06-27

**Authors:** Albert Manero, Peter Smith, Amanda Koontz, Matt Dombrowski, John Sparkman, Dominique Courbin, Albert Chi

**Affiliations:** 1 Limbitless Solutions, University of Central Florida, 4217 E Plaza Drive, Orlando, FL 32816, USA; peter.smith@ucf.edu (P.S.); mattd@ucf.edu (M.D.); john.sparkman@limbitless-solutions.org (J.S.); dominique@limbitless-solutions.org (D.C.); 2 Department of Sociology, University of Central Florida, 4000 Central Florida Blvd, Orlando, FL 32816, USA; Amanda.Koontz@ucf.edu; 3 Division of Trauma, Critical Care & Acute Care Surgery, Department of Surgery, Oregon Health and Science University, 3181 SW Sam Jackson Park Rd, Portland, OR 97239, USA; chia@ohsu.edu

**Keywords:** COVID-19, 3D printing, PPE, social networks

## Abstract

The SARS-CoV-2 (COVID-19) pandemic has provided a unique set of global supply chain limitations with an exponentially growing surge of patients requiring care. The needs for Personal Protective Equipment (PPE) for hospital staff and doctors have been overwhelming, even just to rule out patients not infected. High demand for traditionally manufactured devices, challenged by global demand and limited production, has resulted in a call for additive manufactured (3D printed) equipment to fill the gap between traditional manufacturing cycles. This method has the unique ability to pivot in real time, while traditional manufacturing may take months to change production runs. 3D printing has been used to produce a variety of equipment for hospitals including face shields, masks, and even ventilator components to handle the surge. This type of rapid, crowd sourced, design and production resulted in new challenges for regulation, liability, and distribution. This manuscript reviews these challenges and successes of additive manufacturing and provides a forward plan for hospitals to consider for future surge events. Recommendations: To accommodate future surges, hospitals and municipalities should develop capacity for short-run custom production, enabling them to validate new designs. This will rapidly increase access to vetted equipment and critical network sharing with community distributed manufacturers and partners. Clear guidance and reviewed design repositories by regulatory authorities will streamline efforts to combat future pandemic waives or other surge events.

## 1. Introduction

At the start of January 2020, information began to flow globally regarding the risk of the SARS-CoV-2 (COVID-19) virus to spread, which was first identified in Wuhan City, Hubei Province, China. By 30 January, the World Health Organization (WHO) had declared the situation an international emergency and, by 11 March, a global pandemic [1]. By 15 April, the global confirmed cases had surpassed two million with 130,000 plus associated deaths [2]. This rapid exponential growth has led to dramatic restrictions to support social distancing efforts, thereby limiting the growth of hot spot clusters that were predicted to drive human-to-human transmission. Limitations for modeling the spread of the virus and identifying accurate parameters have yielded challenges in creating policy and procedures. The result has been dissimilar responses across the United State of America collectively and globally.

The rapid surge of cases has placed strain on the global supply chain for medical equipment, personal protective equipment (PPE), and raw materials. The extent of this strain modeled far exceeded the available emergency room beds if left unmitigated [3]. In the United States, concerns for adequate supplies of ventilators have ranged in the millions [4]. Distribution and resource management have been significant challenges. At the present time, the estimates for available ventilators in the United States range between 60,000 and 160,000 [5]. Estimates for supply needs of N95 masks include 2.6 (non-ICU) and six (ICU) changes per patient per day, which could be in excess of 50 million when combined with outpatient and emergency room visits [6]. Surgical masks, which have also been in short supply, are estimated to be in demand by a total of 10 (non-ICU) and 10 (ICU) changes per patient per day, which would be in excess of 150 million total masks [6]. These needs would not include nearly a billion per month for the general public [6] nationally if mandatory masks become routine.

Hospitals around the United States have different capacities reflecting the composition of their communities. The open database [3] of hospital capacity highlights both the ratio of total beds to adult population and the ratio of senior citizens (age over 65) in the population. There have been estimates of an average 15% hospitalization rate [6], which has disproportionately been represented by senior citizens.

Limitations in the availability of sufficient PPE supplies in Italy were considered to be drivers in health workers contracting the virus [7,8]. These concerns have been met by hospitals calling for reusing PPE [9] and donations from community members. These calls have been focused on masks, due to limited N95 availability, along with face shields. Governments began to call for the use of masks, both commercial and homemade production, for individuals to reduce potential droplet spread [10,11]. This need has strained the global supply chain and has had significant influence on availability for hospitals. With much of the global supply chain of equipment originating in China and the simultaneous needs by countries around the world for the same critical products, the availability for the estimated millions of health care works has been challenging [12,13,14]. Recent efforts [15,16] to qualify decontamination methods for the supplies are underway and suggest that decontamination cycles may be permissible.

The immediate response due to equipment shortages and reduced global production has been an all call of community citizen support, corporate manufacturing pivots, and a push for open source designs with standardized production methods to fill the critical time gap. While startup times for traditional manufacturing may take months to change production runs, additive manufacturing can pivot for real time, short-run production. Preemptively developing and maintaining social infrastructure may increase effectiveness in addressing surge demand, as embedded social networks encourage a sense of obligation, trust, and accountability [17]. Social infrastructure is needed in tandem with physical production capacity, contributing to a knowledge chain that enables resource sharing, with resources encompassing materials, human capital including human skill and expertise, and synergistic collaborative partnerships.

This article examines the role of additive manufacturing to expedite the response to support medical institutions. Partnerships that have formed unified production coalitions or collaborative design networks, utilizing 3D printing, are discussed. The strengths and limitations for implementing designs for medical devices and accessories in a distributed manufacturing network are highlighted. The article provides recommendations and reviews the issues surrounding deploying additive manufacturing during surge events, including regulation, design sharing, and ensuring quality. Recommendations are introduced for establishing new community networks and leveraging existing networks in order to rally communities’ corporate and citizen support for pivoting during times of crises. The manuscript looks to highlight the role of academic, corporate, and municipal organizations to utilize 3D printing to support both local and regional needs for design and distributed manufacturing, while encouraging collaboration, appropriate regulation, and preemptive planning for future surge events.

## 2. Discussion

The response to this unique challenge surrounding the COVID-19 surge response is built on lessons learned from establishing 3D printed prosthetic networks and their integration with medical institutions [18,19,20]. Similar challenges regarding adequate regulation, sustaining coalition efforts, production methods, quality control, and design sharing are present. Solutions and best practices learned are leveraged to develop recommendations focused on COVID-19 related distributed manufacturing.

### 2.1. Regulation

The Health and Human Services Department issued a notice and declared a public health emergency on 4 February 2020, identifying a significant potential to affect national security or the health and security of United States citizens living abroad [21]. This declaration gave the authority to the Food and Drug Administration (FDA) to issue Emergency Use Authorization (EUA) for in vitro diagnostics for detection and/or diagnosis of COVID-19 infections. On 2 March, the Health and Human Services Department extended the declaration to include an EUA for personal respiratory protective devices. These declarations enabled (i) the emergency use of an unapproved drug, an unapproved or uncleared device, or an unlicensed biological product or (ii) an unapproved use of an approved drug, approved or cleared device, or licensed biological product [22].

This declaration effectively paved the way for distributed manufacturing for a variety of protective devices and established relaxed parameters for manufacturing and labeling criteria. Specific authorizations for individual PPE were then issued.

Additional emergency guidance for both the approved National Institute for Occupational Safety and Health (NIOSH) filtering respirators and Non-NIOSH-approved filtering respirators was issued on 2 and 24 March. This opened up supply options to accommodate the shortage. Specific to face shields, which have been at the center of the distributed manufacturing efforts, an official letter of authorization was not issued until 3 April 2020 [22,23]. The weeks proceeding had seen rapid viral outbreak growth and a reduction of corporate production. That time period saw a push by manufacturers, makers, and designers working to create on-demand equipment; though questions regarding the regulations still remained.

At the end of March, the FDA provided answers to questions regarding 3D printing for equipment being produced for the COVID-19 response, emphasizing the need for creative and flexible approaches and the limitations of the manufacturing techniques [24]. Without these emergency authorizations, the standard requirements for new manufacturing methods or designs of medical devices would require Institutional Review Board (IRB) governance. Establishing such studies may have long review times before testing or implementation could even begin. There was a cascading delay in starting production to fill shortages at hospitals or strengthen supply chains. This was due in part to local stay at home orders going into effect to depopulate spaces and the limited communication of the regulatory framework. Many institutions and universities struggled to implement distributed manufacturing plans, and laboratories remained closed due to concerns about human safety and production liability. Additive manufacturing has advantages in quickly pivoting or iterating designs, but production for large volume takes a considerable amount of time. Efforts to communicate “as-is” distribution or to execute waivers of liability from the hospitals limited the effectiveness of the response, while under the FDA’s guidelines, manufacturers had additional provisions as long as they were following the Conditions of Authorization established, post-EUA announcements.

### 2.2. Coalitions

The community’s efforts to re-supply hospitals focused on solving different shortages and bringing together enough micro-production sites to have an aggregate support for the real-time need. The FDA’s leadership with the Department of Veterans Affairs (VA) and National Institutes of Health (NIH) [25] provided overarching guidance for design reviews, best practices, and level of comfort for institutions made hesitant by the difficulty in navigating the regulation landscape. This discussion highlights some of the significant collaboration and implementation for a variety of equipment pieces made available.

In order to establish and maintain strong community coalitions, multiple factors are necessary. While a key factor consists of infrastructure (the basic physical structures, facilities, and utilities), the health of the community also rests on social networks. This requires the development of social infrastructure, or a supporting social network of informal associations that provides the opportunity for and gives power to interaction between community members, stakeholders, specialists, and neighborhood associations. Such a network, when embedded into a community, supports “concrete personal relations and networks of relations” that contribute to building trust, establishing expectations, and both creating and enforcing norms [17]. In the context of 3D printing for COVID-19 related medical supplies and equipment, organic networks developed around certain affinity groups including manufacturers in brand ecosystems where trust and communication had been previously established.

Social capital is based on reciprocal relationships that contribute to community development, gaining esteem in a field, and increasing the ability to contribute to the marketplace and production [17,26,27,28]. This knowledge chain can then be more quickly accessed and utilized in times of urgent needs, as associations can contribute to production systems, pivoting and gaining resources for their own changing needs, through knowledge of their creative and physical capabilities. Several coalitions made significant contributions on a national scale by bringing together distributed manufacturing into a focused effort.

#### 2.2.1. Identifying Supply Shortages

A challenge that occurred during the pandemic was how to filter large sets of information for identifying authentic and real-time needs. GetUsPPE [29], an organization focused on connecting the needs of medical providers to community based partners, was able to tabulate and map across the United States. This connection point supported engaging community manufacturers and those with surplus supplies, cataloging approved designs, and engaging volunteers locally for ensuring reliability and mitigating additional risks.

This article focuses on the role of additive manufacturing and community networks’ support for distributed manufacturing. While traditional manufacturing provides long-term capacity to solve supply chain issues, additive manufacturing is unique in the ability to solve short-term time-sensitive shortages. It is important to note that the scale and speed of traditional manufacturing, once established, is critically important. In the United States, car and medical device manufacturers worked to pivot and produced and distributed significant quantities, in the millions, of face shields and other PPE [30,31].

#### 2.2.2. Unified Production Coalitions

Led by the Department of Defense, the National Security Innovation Network (NSIN) [32] brought together additive manufacturing specialists from around the globe. The coalition focused on identifying idle 3D printers at individuals homes, universities, and corporations that could be re-purposed for distributed manufacturing. Their efforts prior to the pandemic have been focused on building networks of innovators that generate new solutions to national security problems. The network supported COVID-19 related challenges for emergency ventilator and face shield design and production.

Prusa3D [33], a 3D printer manufacturer, has developed and validated the Prusa PRO Face Shield design. The design has been provided as an open source resource globally. The company has printed and donated almost 200,000 shields by June 2020 focused on the Czech Republic’s medical workers. Prusa provided guidelines for sanitizing 3D printed parts with validation from Czech laboratories and hospitals to support the community-led efforts. Their work to establish online community networks has supported locally and regionally focused efforts around the globe.

Formlabs [34], a 3D printer manufacturer, worked to validate designs to support production for diagnostic equipment, medical devices, and accessories including face shields, face masks, mask extenders, nasal swabs, and ventilator splitters. Formlabs’ support network has rallied more than 3000 volunteers to leverage distributed manufacturing equipment globally to connect with medical institutions with needs for both medical devices and accessories.

Stratasys [35], a 3D printer manufacturer, brought together corporate and education partners with the capacity to produce face shields rapidly for distribution to a national coalition of hospital and medical sites in need. With over 100 operating production sites, the coalition distributed over 100,000 3D printed shields to more than 200 medical sites nationally, with traditional manufacturing adding to these numbers significantly. The coalition worked with the joint partnership between the FDA, Department of Veterans Affairs, and Department of Health and Human Services to have designs and production methods approved for use, helping to streamline all distributed manufacturing sites on one consistent design. Designs supported by the coalition include face shields and nasopharyngeal swabs. The coalition established a network through GrabCAD’s platform to coordinate and unify the distributed manufacturing.

Glowforge [36], a 3D laser printer (cutter) manufacturer, has rallied its user base of makers and manufacturers to produce millions of earsavers, small accessories that relieve the pressure on the ears of a person wearing surgical masks. With the need to wear masks at all times on shifts, hospitals began to see the physical challenges associated with this. These devices can be rapidly produced, via 3D printing and laser cutting, and have represented one of the most in-demand accessories to support medical staff. This small accessory allows greater flexibility for mask size fitting individuals, which has supported the challenges due to the limited supply chain. The requirements for manufacturing tolerance and fit for these devices are much lower than some of the other equipment being requested by hospitals, which has led the maker community to be able to step in and provide significant support locally and nationally with over 500,000 shipped in April.

#### 2.2.3. Design Work, Challenges, and Resource Sharing

Educational institutions from around the United States have rallied to support their local communities through design work for face shields and other PPE or accessories, with manufacturing techniques including 3D printing and traditional injection molding [37,38,39]. By providing open source design files and instructions, these local networks have supported innovative design iteration and community focused engagement. Particular success was achieved for face shields and accessories. Sharing clear manufacturing and validation instructions and utilizing the NIH 3D Print Exchange amplified the efforts.

HP [40] took a unique corporate approach to curate a selection of designs available to manufacturers and makers to be able to support 3D printing for medical devices and accessories. This included face shields, various types of conforming masks, and accessories such as mask adjusters, hands-free door openers, and wrist coverings or gauntlets. The corporation worked with customers and designers to optimize and host the designs for additive manufacturing and for specific global applications. Specific support for design and validation for more complex, higher risk, equipment such as field respirators and ventilator components has been critical to identifying innovative ways to back-fill supply if hospitals face catastrophic shortages of equipment.

The “CoVent-19” Challenge [41] has been ongoing nationally throughout the pandemic, first proposed by Massachusetts General Hospital and sponsored by Stratasys, Ximedica, Valispace, HackFund, and Yelling Mule. Early on in the pandemic’s spread, fear revolved around limited ventilator devices not being able to support the full patient load, though the specific application of ventilators has been questioned and debated as new information becomes available [42,43]. This challenge was inspired by the crisis in Italy resulting in a significant deficit of ventilator valves available and a Fablab that began to print valves directly for a hospital, with additional support of a 3D printer manufacturer Isinnova [44]. While parts were made available to the hospital, there was considerable concern for liability and legal challenges due to copyright and patent law that has limited the application. In similar fashion, HP provided very clear legal liability waivers when providing designs for the public to manufacture in a distributed manner.

### 2.3. Strengths and Limitations

Each device or accessory that has been explored for distributed community manufacturing has had strengths and limitations for application. Additive manufacturing in particular has many strengths including low short-run manufacturing, highly customizable geometries, and the ability to distribute manufacturing locally for replacement parts. 3D printing is subject to high variability, between machines and also between individual prints. The quality of production is also subject to the printing raster orientation, fiber thickness, and load cases in application [45,46]. Some limitations are manufacturing material dependent, with lower cost printers expected to have higher variability and limited production materials. Different machines use dissimilar techniques for producing a polymer based 3D printed part. Fused Deposition Modeling (FDM) is perhaps the most commonly available, but the extrusion of material leaves large porosities in the final part. This porosity, coupled with variability in the production process, can lead to significant challenges regarding durability and difficulties in clearing the final part for environments that require a higher degree of sterility. Selective Laser Sintering (SLS) uses a powder bed and laser for growing the modeled part. Resin printers often have the highest resolution of 3D printers, with the pixel resolution between 50 and 100 microns and the ability to produce a wider range of material properties.

#### 2.3.1. Face Masks

Face masks have been central to the conversation around distributed PPE production, with 3D printing being a key manufacturing method [47,48]. In the United States, there have been significant shortages of supplies for hospitals and citizens and efforts to support manufacturing using 3D printing [49,50]. The strengths of this application have included the ability to custom fit each design to the person intended to wear the device, using digital software tools, and some of the exterior durability. Challenges have included that the process requires more design effort per individual production to use stereophotogrammetry and digital tools to ensure an accurate fit. These types of masks when manufactured traditionally still require fit checks, breathing checks, and some degree of evaluation to know that the seal is working. The manufactured parts still require a filter material to be effective. Many 3D printer materials are not ideal for long-term skin contact; disinfecting printed materials can be challenging; these challenges can be exacerbated in a warm humid environment due to the environment or, in the case of a mask, the donee’s breathing. For these reasons, distributed additive manufacturing for face masks has been limited.

#### 2.3.2. Face Shields

**Figure 1:**
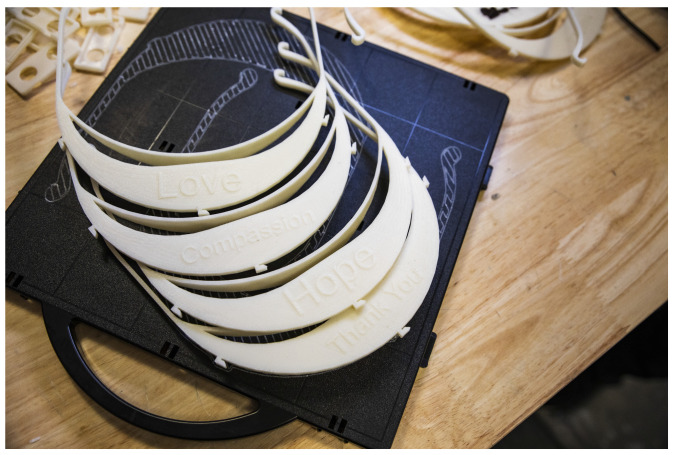
3D printed face shield visor manufactured in out laboratory to support national coalition efforts. Design: Stratasys’ three hole punch spacing visor. Machine: Fortus 250 mc. Material: acrylonitrile butadiene styrene.

Face shields have been one of the coalitions’ success points in this pandemic. Visor designs were able to be streamlined early on for manufacturing on a 3D printer with limited support materials, though a preference for ABS plastic or more sophisticated bio-compatible or nylon materials has excluded some desktop or hobbyist machines. 3D printers were not well suited for the shield component of the device, and the success of the coalitions was attributed to corporate partners being able to procure large quantities of clear shielding materials to pair with the distributed network’s final visor parts. Having a clear coalition leadership also provided mitigation to questions regarding liability or distribution, as central support agreements by the coalition allowed community-led efforts to focus on keeping the machines and production running without the encumberment of legal challenges or constant customization for each production run. These factors have enabled the device to find greater success than otherwise possible. The FDA’s EUA announcement cleared the way for distributed additive manufacturing being ready for large-scale deployment as a stopgap measure before traditional manufacturing supply chains could catch up to demand. A 3D printed face shield produced in our laboratory is presented in Figure 1.

#### 2.3.3. Ventilators

Ventilators have been one of the most challenging items to design for using additive manufacturing. While initial results had proven promising, there are a number of limitations that keep the method from being implemented across the world. The challenge with mechanical ventilator parts being produced in this manner is that the loading conditions require good seals and durability, though filters and additional sealant can certainly support these efforts. The load case requires high durability under cyclic loading, and manufactured parts with higher variability are more prone to failure when exposed. The liability in such critical cases for these experimental parts has also limited the application, though much promise remains for hybrid manufactured devices currently in evaluation across the world. Assembly with traditionally manufactured parts is a necessity, which may be difficult to source distributively and requires additional operator specification including robust testing capabilities.

Progress is being made towards 3D printing of ventilator components and accessories. Designs from institutions have progressed to submissions to the FDA for EUA and animal studies [51,52,53]. To prevent complications including barotrauma, important design considerations and extensive validation need to be conducted. Features including adjustable Positive End Expiratory Pressure (PEEP), Peak Inspiratory Pressure (PIP), and inspiratory timing must be considered. This type of design requires specific medical oversight to address challenges beyond the mechanics. The lack of these design features and oversight might result in harm to patients. The CRISISvent [52] has been designed for adjustable positive end expiratory pressure and allows for spontaneous breathing. These critical design features are also sensitive to manufacturing precision and are an excellent example of the need for collaboration between designers, manufacturing experts, and medical practitioners.

#### 2.3.4. Nasopharyngeal Swabs

A key success metric for states has been the volume of COVID-19 reverse-transcriptase polymerase chain reaction testing able to be processed daily. One limitation has been in the supply chain of the physical swab kits required to collect samples from patients, which are considered an FDA Class 1 exempt device [54]. A coalition of manufacturers, institutions, and medical sites has been launched by Beth Israel Deaconess Medical Center [55]. The complexity of design validation and testing was accomplished in a matter of weeks, and multi-phase clinical evaluation was completed. Design information and point of sale for testing kit items are now available. This type of distributed design and manufacturing collaboration brought together a variety of expertise from industry and academia. This provides a valuable case study to model sustained coalition cooperation for future surge events.

#### 2.3.5. Accessories

**Figure 2:**
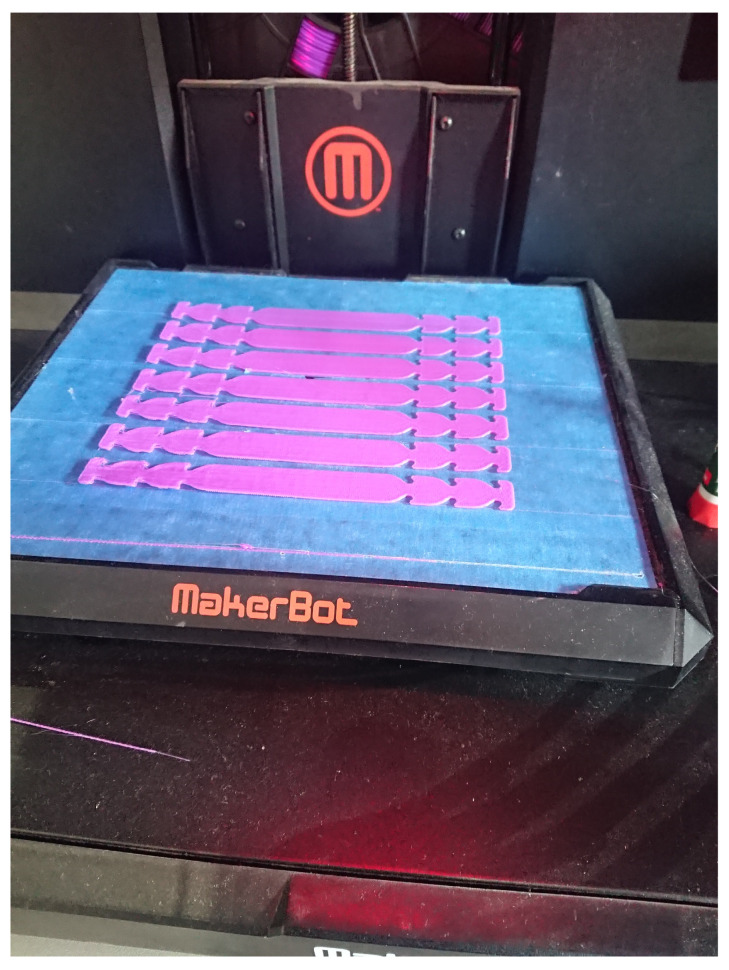
Example 3D printed mask extender set, or earsaver, manufactured to support local hospital needs on a desktop-style 3D printer. Design: disposable ear relief strap [56]. Machine: MakerBot Replicator. Material: polylactide thermoplastic.

Equipment accessories have been perhaps the greatest area for hobbyist, community based manufacturing, and corporate groups to partner with, which require limited assembly to solve meaningful challenges in the supply chain for hospitals. Earsavers, or mask extenders, have been able to be produced in a variety of different materials and can be produced more quickly due to limited complexity and size. An example of 3D printed mask extenders produced in our laboratory is presented in Figure 2.

This fault-tolerant design can be produced by both laser cutting and 3D printing on hobbyist machines. As these devices are designed to be more readily disposable, many challenges were able to be mitigated. For reducing the strain of wearing surgical masks or adapting ill-fitting masks to a larger range of face shapes, the device has been highly effective. Energized hobbyist and craft makers have supported significant manufacturing throughput to support medical workers, as well as civilians. Other accessories have seen limited application at scale, but solve local challenges.

### 2.4. File Sharing and Security

Distributing design files across the Internet have made rapid prototyping and local production feasible in a variety of fields including prosthetics and medical devices ([18,19,57,58]). There are a number of repositories for 3D design files including Thingiverse, GrabCAD, Prusa Printers, and the National Institutes of Health (NIH) 3D Print Exchange. As of 2019 [59], Thingiverse, the largest of these repositories, had two million registered users and cumulatively more than 340 million downloads. These platforms range in levels of curation from highly open models to file submissions reviewed prior to hosting. While the open model allows for rapid derivations and collaboration, this format proves difficult for designs to be used in the medical field where standardization is required. The Stratasys-led coalition for COVID-19 face shield production utilized the GrabCAD printing platform, which allowed the leadership to vet each production site and assign specific files to each order. Submitted files related to COVID-19 on the NIH established platform were reviewed and curated through a partnership with the FDA, VA, and America Makes prior to receiving a distinction of “Clinically Reviewed”. For community based distributed manufacturing to be successful, it is a priority to have well vetted and standardized files that are easily accessible where file security and authenticity are paramount.

## 3. Recommendations

The cascade of challenges associated with fighting the COVID-19 pandemic has revealed a number of vulnerabilities in our medical readiness. While there are still unknowns regarding this virus and its long-term effects, seasonality, and possible mutation, there are several actions that can be taken to improve the systems’ resilience in the midst of surge demand and global supply chain challenges and conflicts.

### 3.1. Hospital Infrastructure

A top priority moving forward is to support hospitals developing infrastructure for design and short-run manufacturing capabilities. While this is not a call for hospitals to become manufacturers, having short-run capacity will support them in the following ways:

(i) Manufacturing and design capacity will allow the design and evaluation for parts and equipment prior to critical need. This will enable hospital staff to review current capacities and identify strategies for frequently replaced or consumed parts. Developing designs for spare replaceable parts prepared prior to critical needs can reduce inventory demands. When surge demands or supply chain issues emerge, hospitals will have standardized and validated designs to engage community and national coalitions to pivot manufacturing needs quickly to fill gaps in the short term. This capacity will also encourage hospitals to take an innovative approach toward the tools and equipment used in the hospital, which may improve surgical instrumentation and use-specific tool situations.
(ii) Emergency manufacturing may be possible during surge events for hospitals with sufficient equipment and design knowledge. For certain equipment items, like face shields, hospitals may be able to have supply stock ready in case of emergency needs to augment traditional supplies. At the start of potential surge events nationally or internationally, short-run manufacturing can be initiated to increase supplies of key components with the support of community networks.

### 3.2. Regulation

This surge event has revealed challenges with quickly pivoting, including regulation and standardized design sharing processes. The FDA’s efforts to bridge the public and private sectors has enabled the NIH 3D Print Exchange to provide clear information on files that have been reviewed for acceptable implementation, following certain production and quality control measures. The review process in the partnership to label 3D models “Reviewed for Clinical Use” during periods of emergency use authorizations provides manufacturers clear and reliable sources for files and production strategies. For these distributed manufacturing networks to be successful for future events, assurances for standardization, liability, and quality control have to be prioritized. Top priorities to increase readiness and distribute manufacturing participation include two key areas.

(i) Having archived reputable designs and instructions will reduce the time to start broad distributed manufacturing efforts, critical to building sufficient supply before the surge. This partnership and print exchange are critical for future surge events, and a dedicated curation of designs and production techniques should be prioritized by NIH and the FDA for rapid implementation.
(ii) Guidelines and training information for local Institutional Review Boards, liability, and risk management should be prioritized to clearly communicate best practices, authorization requirements, and safety guidelines. This will reduce the time for coordinated efforts to begin for academic institutions, municipalities, and corporate manufacturers and ensure that uniform protocols are implemented.

### 3.3. Leverage Existing Communities

There is capacity and interest in coordinate support efforts from several existing communities including local, educational, and defense oriented networks. In recent years, the availability of additive manufacturing has made its way to local libraries, makerspaces, and other municipal centers. The capacity of these fabrication sites may be appropriate to tackle local challenges during times of community stress, and the technical design capacity may be able to be leveraged to support medical institutions for prototyping and project based problem solving.

#### 3.3.1. Education Systems

State and university leadership are calling upon their collective knowledge of professors, students, researchers, and staff to find innovative ways to support healthcare workers within their communities, building upon the production, research, multidisciplinary collaboration, and makerspace resources. With significant resources well distributed across the country, it is imperative to find methods to activate these resources appropriately, strategically, and with great care. For many universities, this innovation has come from makerspaces, research, and fabrication labs housed within the areas of engineering and health sciences. Due to the unique challenges of the COVID-19 pandemic, universities have also looked to the liberal arts areas to assist in areas including textile fabrication. It is important to enter this support role with care and caution, with a well communicated expectation of standardization and the application in a hospital environment. To create robust education-led response networks, communication and multidisciplinary collaboration will be required.

**Figure 3:**
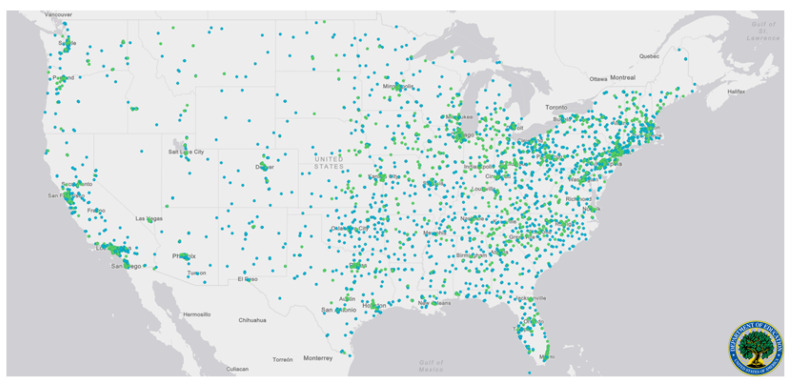
A national map identifying higher education institutions across the United States. Blue dots are used to represent public universities, and green dots represent private not-for-profit universities. Data and mapping made available by the Department of Education [60].

Geographically, the United States’ university system of public and non-profit private institutions is well distributed. With significant state and federal infrastructure committed to university research and student success, this network of potential distributed fabrication sites during surge events could be activated. The distribution of these institutions is presented in Figure 3. The distribution of these institutions and their available equipment provide a strategic resource for communities, if networks can be formalized. While COVID-19 has produced a more uniform disruption and surge, the proposed networking of institutions will be especially effective during localized disaster situations where other non-affected nodes can be activated to support afflicted locations. There are particular applications during localized or regional weather events, such as during hurricane season.

With universities having a degree of informal internal networks with professor-led research equipment at each institution, having a clear activation plan prior to surge events will reduce the time required to support afflicted local nodes. Participating institution protections and clear standards will be critical to have in place. Professional societies, which commonly engage with chapter sites across a wide range of institutions, may be uniquely positioned to support the coordination alongside institutional leadership. While the goal of the proposed network is not to transform universities into large-scale manufacturers, there is an opportunity to support short-run manufacturing, resource sharing, and rapid prototype design to support localized disaster or surge events. As seen in the COVID-19 initial surge, which had a significant impact on the supply chain, localized distributed manufacturing with 3D printing has been able to fill a critical time-sensitive gap while traditional manufacturing at a large scale requires time for setup and pivot.

#### 3.3.2. Defense Industry

In a report from the Inspector General’s Office for the United State Department of Defense, it was found that at least 81 Military Service Depots maintain facilities that use additive manufacturing, to reduce costs and improve readiness [61]. The same report describes the use of Rapid Fabrication via Additive-Manufacturing on the Battlefield (R-FAB) units that are deployed along with the U.S. Army to locations including Germany, Thailand, Japan, and South Korea, ensuring access to what the military is considering an invaluable resource. The capacity for critical real-time manufacturing ensures sustainability. The military has similar infrastructure for supporting design files. The Navy maintains the Joint Technical Data Integration database, which is a database that includes technical manuals, training, and approved part design files for additive manufacturing that can be shared across services. The Air Force is actively running the Reverse Engineering and Critical Tooling (REACT) Laboratory. This group is reverse engineering parts and developing plans for replacement part designs that can be readily manufactured in-house or in the field.

(i) It is our recommendation that during extreme times of surge disaster events that a synergy between the National Institutes of Health and the Department of Defense link critical health oriented design files and potential disaster zone short-run manufacturing to prevent mass casualties due to insufficient critical care equipment. Medical institutions can leverage this knowledge and preparedness to increase response time and coordinating local and national networks supporting relief and supply production.

### 3.4. Establishing Community Networks

The importance of establishing these networks, in the case of addressing surge demand, is to develop readiness and action plans prior to the supply shortage. The ability to establish and maintain contacts that build and diversify social capital in an ongoing manner is as important as the physical production capacity. The authors propose three considerations.

(i) Evaluate community resources: In order to support the hospital infrastructure, a mesh network of distributed manufacturing facilities must be developed. This is complex as it extends beyond the available equipment. There is a need to evaluate what type of equipment can be useful in a crisis for specific types of productions, what facilities or networks in local communities already supply this equipment, how it can be leveraged to maximum efficiency and flexibility, and what parts or hardware are susceptible to shortfall in the case of a crisis. The needs must then be evaluated for the ease of manufacturing through tools available locally including maker spaces and other corporate or educational manufacturing facilities. These facilities must be vetted for the quality of the equipment, supporting the right materials, and for the ability to deliver, house, and manipulate the appropriate materials. This database should be handled by a central organization that should keep track of machines, materials, and expertise.
  Informal networks and organizations that are created to solve one particular problem can come to remain after the initial need, becoming more established and able to address a broader scope of issues [17]. This is an important consideration in response to COVID-19 in two directions, in that production services can contemporaneously look to informal community networks to further develop social capital that enables a more effective and resilient response, while the informal networks that became established during this time should aim to be maintained as a form of social infrastructure to meet future demands.
  Empowering an equitable, embedded social infrastructure will entail (1) acknowledging power differentials (economic, physical, and human capital), (2) determining stakeholders (e.g., small and mass producers, investors, at-risk community members), and (3) developing trust in the social network. This process of embedding social infrastructure therefore also includes transparently sharing resources and getting commitment from stakeholders, and more specifically determining the beneficiaries. Beneficiaries, in this instance, relate to the positionality of organizations and individuals. Creating a knowledge chain that includes those who are likely to be in need of services, who offer interpersonal services (e.g., nurses), and those on the production side will have differing knowledge of and impact on production and distribution. Therefore, involving a variety of stakeholders in the creation of community networks can greatly benefit the development and embeddedness of social infrastructure, creating higher buy-in and ability to activate social resources quickly.
(ii) Support a repository of vetted designs: The community must be supported by vetted infrastructure that provides access to designs, keeps track of distributed manufacturing resources, and can aid in logistical support. The design of the parts being created should be handled by a central organization that not only keeps track of the available infrastructure and provides vetted designs. These designs should, when possible, be pre-vetted and approved by governing organizations. Effort should also be made to support IP and ownership of industrial and medical designs; permission should be sought from the patent holders for any parts that are exclusive to one organization, but saving lives should be the primary focus of such a repository of designs. This may require government leadership to allow emergency production to support distributed manufacturing in short-term emergency situations.
  This distribution process can be expedited through the development of social infrastructure, as network members can establish norms of reciprocity, sanctions that uphold standards of emergency response, trust, and informational channels. The obligation of saving lives, as a primary focus, can establish expectations and direct focus to identifying resources available to each party even by sharing needed production resources in exchange for needed expertise.
(iii) Encourage participation and protect participants: Concrete rewards and recognition must be incorporated to encourage continued support of the coalition. Developing social infrastructure that supports informal associations that enable community members, stakeholders, and specialists to participate smoothly not only requires community support for vetted files and logistical support, but participants in this ecosystem must feel safe, helpful, and rewarded. In an effort to expand the reach of such an enterprise, the authors suggest technical, professional organizations consider leading in a multi-tier local and national approach. Organizations and municipalities can leverage the open database [3] to establish guidelines for distributed manufacturing volume appropriate for their local community. Establishing the clear communication of stakeholders and recipients, in particular in the local network, will strengthen participation.
  Investing in social capital can encourage a sense of shared responsibility through civic engagement and, when based on authentic participation, trust from participants. This encourages decision-making from the ground-up to address “local” needs, but needs to be done in such a way that a process is in place for strategic activities, helping to create a power balance. Additionally, while not directly connected to economic capital, the development of reciprocity can encourage further investments, expanding potential monetary benefits to involved parties. This, in turn, increases the strength of the community network and emergency response abilities. Such social networks can then be seen as a resource to an individual or corporation, increasing capacity to meet certain ends otherwise not possible. This synergistic production process is therefore based on the relations amongst members, regulating production and services as social capital “allows” certain activities and actions to happen because it is also understood that only certain actions are acceptable. Establishing norms that expand beyond institutional regulation can encourage the trust and social regulation during a surge that help to serve as a stopgap for safety and public information trust. The mesh network, or mesh of networks, can be uniquely positioned to support local surges or disasters that limit a local response.

## 4. Conclusions

For society and medical institutions to be better prepared for future surge pandemic events or other natural disasters, establishing community distributed manufacturing and care networks is of high priority. Having pre-validated design files that can be quickly short-run manufactured to solve time-sensitive shortages for critical care needs will enable facilities to provide the highest level of patient care while protecting their medical personnel. To support these efforts, clear guidance from regulatory bodies and curated design repositories are essential. Establishing and building on the coalitions and networks that were initiated during COVID-19 Surge 1 will be critical to mitigating future risks and shortages. With strong community engagement and support networks, a more robust network can form that will be even more effective when localized surge or disaster events occur while more distant network nodes can back-fill supply. Municipalities and educational institutions can be leveraged to establish strong and flexible networks of human capital and production capacity, leading to shorter and less frequent waves of disaster response.

## Abbreviations

The following abbreviations are used in this manuscript:

COVID-19	SARS-CoV-2 Virus Disease
DoD	United States Department of Defense
EUA	Emergency Use Authorization
FDM	Fused Deposition Modeling
IRB	Institutional Review Board
NIH	National Institutes of Health
NIOSH	National Institute for Occupational Safety and Health
PEEP	Positive End Expiratory Pressure
PIP	Peak Inspiratory Pressure
PPE	Personal Protective Equipment
R-FAB	Rapid Fabrication via Additive-Manufacturing on the Battlefield
REACT	Reverse Engineering and Critical Tooling
SLS	Selective Laser Sintering
